# Efficacy of onabotulinumtoxinA treatment in episodic migraine

**DOI:** 10.3389/fneur.2024.1459767

**Published:** 2025-01-06

**Authors:** Jasem Al-Hashel, Raed Alroughani, Malak Almojel, Samar Farouk Ahmed

**Affiliations:** ^1^Department of Neurology, Ibn Sina Hospital, Safat, Kuwait; ^2^Department of Medicine, Faculty of Medicine, Kuwait University, Safat, Kuwait; ^3^Division of Neurology, Amiri Hospital, Sharq, Kuwait; ^4^Department of Medicine, Amiri Hospital, Sharq, Kuwait; ^5^Department of Neurology and Psychiatry, Minia University, Minya, Egypt

**Keywords:** episodic migraine, botulinum toxin-A, migraine-specific quality of life, productivity and activity impairment, headache

## Abstract

**Background:**

OnabotulinumtoxinA (BoNT-A) is approved as a prophylactic treatment of chronic migraine (CM) only. We aimed to assess the efficacy and safety of BoNT-A in the treatment of episodic migraine (EM).

**Methods:**

This is a prospective study included migraine patients, aged 18–65 years, and completed 1 year treatment with BoNT-A. Patients received 4 courses of BoNT-A treatment. Patient’s headache was assessed by headache diary at baseline, and before every injection. Migraine Specific Quality of Life Questionnaire (MSQ) and work productivity were collected at baseline and in their last visit. Adverse events (AEs) were reported.

**Results:**

The study recruited 210 patients. Between baseline and the final visit, there were a significant reduction in migraine days, analgesic consumption days, and headache severity (9.54 ± 1.70 versus 4.58 ± 2.77, *p* < 0.001), (8.47 ± 1.49 versus 2.98 ± 0.21, *p* < 0.001), (8.37 ± 0.72 versus 2.54 ± 0.18, *p* < 0.001), respectively. BoNT-A treatment reduced the mean number of missed hours from work and daily activities over a 7-day period (4.63 ± 2.39 versus 6.26 ± 2.04, *p* < 0.001); (2.24 ± 3.30 versus 3.94 ± 3. 45; *p* < 0.001). Treatment with BoNT-A significantly improved the MSQ scores at last visit versus baseline visit, MSQ Role Function-Restrictive (51.55 ± 29.12 vs. 26.89 ± 17.42; *p* < 0.001), MSQ Role Function-Preventive (56.07 ± 24.73 vs. 30.64 ± 15.25; *p* < 0.001), and for MSQ Emotional Function (76.47 ± 115.29 vs. 35.12 ± 20.83; *p* < 0.001). Fifty-four patients (14.4%) experienced mild and short-lasting AEs.

**Conclusion:**

BoNT-A is an effective and well tolerated therapy in the prophylaxis of EM. It improved MSQ and WPAI.

## 1 Background

Headache is one of the most common reasons that push the patient to visit the primary care (1). It is the second leading cause of years living with disability (2). Migraine is the second most common primary headache disorder and accounts for most of the headache related disability and clinic visits (3). Migraine prevalence is around 10% worldwide and it imposes a huge burden on society (2, 3). International Classification of Headache Disorders, third edition (ICHD-3) has clearly defined diagnostic criteria of migraine (4). According to ICHD-3 criteria, patients with migraine can be further diagnosed with chronic migraine (CM) when having 15 or more headache days per month with at least 8 days meeting ICHD-3 criteria for migraine with or without aura (4). Although there are not any specific diagnostic criteria for episodic migraine (EM) in ICHD-3, the term refers to those individuals with fewer than 15 headache days per month, in contrast to chronic headache (CH) (4). EM was reported to be 23% and chronic headache 5.4% among Kuwaiti population (5).

Preventive treatment benefits many people with migraine, in terms of not only reduction in frequency of migraine attacks, and use of analgesic medications (6) but also improvement of quality of life (7).

Migraine prevalence is highest during the most productive years of life, leading to restricted activity and decreased productivity (6). EM had a significant negative impact on activity of daily living, schooling/employment, and social occasions among migraine patients (5, 8). Disability associated with EM is like that seen in CM (9).

Migraine-Specific Quality of Life (MSQ) scores shown to be significantly lower among patients with migraine headache days per month ≥ 4 (8, 10). Workdays loss may have a significant negative impact on the overall career of subjects with migraine (6).

Food and Drug Administration (FDA) approved the preparation of OnabotulinumtoxinA (BoNT-A) as preventive therapy of chronic migraine based on two large clinical trials (11, 12). Analysis of previously available data for the use of BoNT-A in patients with EM failed to provide adequate evidence for its use in subjects with episodic migraine (13).

The aim of our study was to evaluate the efficacy, tolerability, and impact on disease burden of the use of BoNT-A in the prevention of episodic migraine.

## 2 Methods

### 2.1 Study design and sitting

This prospective study was conducted. It was run in a specialized headache clinic in Ibn Sina hospital which is a tertiary hospital in Kuwait. Self-assessment questionnaires were used to assess the treatment outcome. The enrollment period was January 2021 to December 2021 followed with 1 year follow up. The study was conducted over 48 weeks. With baseline data assessed retrospectively from the month before treatment commences. There was a total of 5 visits on Day 1 and at Weeks 12, 24, 36 for medication and assessment and at week 48 for assessment.

### 2.2 Study population

Eligible patients for the study, male or female patients aged 18–65 years who are diagnosed with EM with or without aura according to ICHD-III (4). They have frequency of migraine headache days less than 15 days over the previous 3 months before initiation of BoNT-A treatment. The study identified migraine patients who had at least three rounds of BoNT-A and completed 1 year follow up. Subjects who can use the headache written diary were included in the study.

The study excluded patients’ diagnosis of another headache disorder including CM, concomitant use of prophylactic treatment for migraine as beta blockers, anticonvulsants, tricyclic antidepressants, or calcium channel blockers. Also the study excluded patients who had previously received botulinum toxin, who had known or suspected drug or alcohol abuse, or those on narcotics, drug overuse headache and with patients with a psychiatric disorders or chronic medical problems, pregnant or breast-feeding patients were also excluded from the study. The study also excluded patients who receive other prophylactic treatment for migraine during the study period. A total of 210 subjects with complete date were identified to the study.

### 2.3 Treatment procedure

Subjects with EM received BoNT-A (BOTOX; Allergan) every 12 weeks (±7 days) for four treatment cycles. BoNT-A injection protocol: Patients with EM received BoNT-A according to the Phase III Research Evaluating Migraine Prophylaxis Therapy (PREEMPT) paradigm (155 U, 31 sites) (11.12).

### 2.4 Data collection

Collected data included gender, age, disease duration, days of migraine per month before treatment with BoNT-A injection, and previous prophylactic therapy. The numbers of the different preventative medications that patients were taking were recorded.

Patients who satisfy the inclusion and exclusion criteria were enrolled for the study after obtaining an informed consent. Patients were trained to keep a headache diary. They were instructed to note down the headache parameters like, duration, number of headache days, severity and number of days on medications. Patients were asked to differentiate between migraine days, with attacks fulfilling the ICHD-3 criteria for migraine (4), and non-migraine headache days, which were not considered in the analysis of this present study. Headache diary was completed daily by participants throughout the study. On the next visit, patient’s headache written diaries were verified, clarified, and documented by the author JYA.

Baseline for the efficacy measures was defined as the frequency of migraine headache days, number of days of analgesic use and name of analgesic, and headache severity during the 30 days prior initiation of BoNT-A treatment. The numbers of days that the patient required to use analgesic for their headache. The patients were asked to fill in the diary for the last month before treatment initiation.

The attack severity using the 0–10 Numerical Rating Scale (NRS) (14) was assessed. The RNS instructs patients to rate their severity of pain during headache attack on a scale from 0 (no pain) to 10 (worst pain imaginable). Disability using the MSQ questionnaire (15, 16) and Work Productivity and Activity Impairment (WPAI) Questionnaire were assessed (17).

MSQL and WPAI questionnaire administered by the clinician at baseline and at the end of observational period were also recorded. Physician provided Arabic version of the migraine-specific quality of life questionnaire (MSQ), version 2.1 (18) to migraine patients to measure the quality of life of the patients with migraines (18). Linguistic validation of Arabic translation was by GlaxoSmithKline Research and Development Limited (GSK) (19). The MSQ version 2.1 is a 14-item questionnaire that measures quality of life (QoL) impacts in three domains. Role Function-Restrictive (RFR) domain includes seven items that measure the functional impact of migraine through limitations on daily social and work activities. Role Function-Preventive (RFP) domain includes four items that measure the impact of migraine through prevention of daily work and social activities. Emotional Function (EF) includes three items that assess the emotional impact of migraine. Raw scores in each domain were computed as a sum of relevant item scores, whereas the raw total score was the sum of all item scores; these were rescaled from 0 to 100, with a higher score indicating better QoL. MSQ the RFR domain is a valuable tool for assessing the functional impact of migraine in chronic and episodic migraine clinical trials (20). All the recorded data were stored in a computerized database.

### 2.5 Outcomes measured

The primary outcome was the change from baseline (30 days prior treatment initiation) to last visit in the migraine days. Secondary outcomes were change in the number of analgesic days and change in the severity of headache (30 days prior treatment initiation). Also changes in in the MSQ and WPAI score as well as safety and tolerability in the last observational visit compared to that at baseline visit. A migraine day was defined as a calendar day with a headache of 4 or more hours’ duration and/or a headache of any duration if acute migraine medication was taken. The primary time point was selected to assess effectiveness after four cycles of BoNT-A had been administered.

Satisfaction with treatment was assessed on numerical scale between 1and 10. One means no satisfaction and 10 means highly satisfied.

The migraine days, number of analgesic days and severity of headache pain were assessed at baseline visit and were evaluated every visit for BoNT-A treatment. Their mean every month was calculated. MSQ and WPAI questionnaires were collected at baseline and in their last visit.

### 2.6 Safety and tolerability

Safety and tolerability were assessed by reviewing the frequency and nature of adverse events (AEs). Patients were advised to report any adverse effects any time during the study period. AEs were determined at each visit through patient self-report, general non-directed questioning also examining the patients’ headache diaries, AEs, discontinuations, and reasons for discontinuation were recorded for each visit. AEs with a reasonable relationship to BoNT-A injections were summarized by overall counts and percentages.

### 2.7 Statistical analysis

Statistical analyses were performed with IBM SPSS Statistics 25.0 software for Mac (SPSS Inc., Chicago, IL, United States). Simple descriptive statistical tests were used to describe the numerical values as means and standard deviation/standard error. Whereas categorical ones were expressed as proportions and percentages. To assess the scores of the subscales of MSQ, each response was coded as follows: always as (1) point, almost as (2), pretty as (3), sometimes as (4), rarely (5), and never as (6). For each subscale, a formula was provided by the designers of the questionnaire as follows: For the first subscale of limitation of patients’ performance, the equation for interrupting normal activity subscales, the equation is (Score-7)x100/35 and for the effect on emotions subscale, the equation is (Score-4)x100/20 and for the effect on emotions subscale, the equation is (Score-3)x100/15. Paired sample *t*-test was used to compare between continuous variables. To prove the main estimator and the relative measure of uncertainty, confidence intervals (CI) was reported or standard errors or both. A significant difference was set to be at *p* < 0.05.

### 2.8 Ethics statement

All subjects gave an informed consent in accordance with the Declaration of Helsinki. The study was performed in observation of the latest version of the declaration of Helsinki [21], and all data was anonymous and protected in accordance with the ethical guidelines of the Council for International Organizations of Medical Sciences [22]. Patient provided informed consent prior to enrollment. All the recorded data were stored in a computerized database.

## 3 Results

### 3.1 Demographics and baseline characteristics

**Figure 1:**
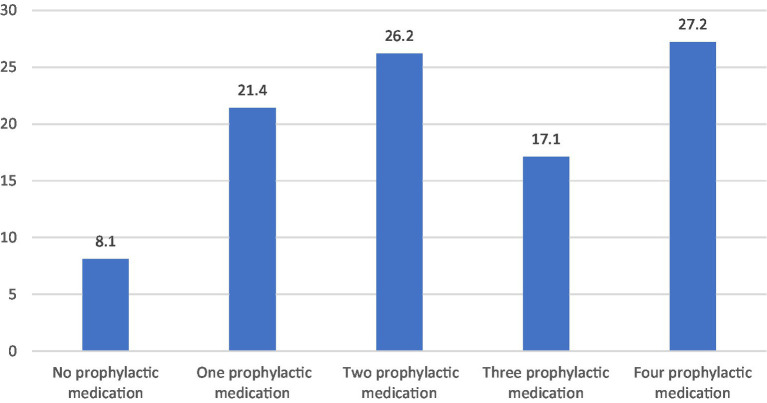
Number of tried previous prophylactic medication.

The principal characteristics of the individuals are shown in Table 1. A total of 210 patients were included in this study, constituting primarily of females (87.1%) with a mean age of 45.04 ± 8.92. The mean ± SD disease duration of patients was 18.40 ± 10.39 years. Majority of the enrolled participants (91.9%) were using prior oral preventive medications; however, they discontinued the preventive medications prior initiation in this study. They used prior prophylactic treatment in therapeutic dose for sufficient period but discontinued due to inefficacy, intolerance or non-adherence. Those who used BoNT-A as first preventive therapy, are patients with high frequent severe episodic migraine who have contraindication to prophylactic treatment or have issue with adherence. Number of tried previous preventive medications was illustrated in Figure 1. The oral preventive medications used most prior to this study were anticonvulsant (43.1%) and antidepressants (30%), followed with traditional medicine (10%), then Beta blockers (8.6%). 8.1% did not receive previous prophylactic treatment. The main indication of discontinuation of prophylaxis in our cohort, reporting up to 66% of participants, is the lack of efficacy.

**Table 1:** Demographic and clinical characters of migraine patients on BoNT-A* treatment (*N* = 210).

Variables	Mean ± SD/Number (%)
Mean age in years	45.04 ± 8.92
Range	23–62
Gender	
Male	27 (12.9)
Female	183 (87.1)
Mean disease duration in years	18.40 ± 10.39
Range	2–44
Previous use of prophylactic medications	
Yes	193 (91.9)
No	17 (8.1)
Cause of discontinuation of previous prophylactic treatment	
Adverse events of previous prophylactic medication	30 (14.3)
Lack of efficacy of prophylactic medication	139 (66.2)
Adherence	15 (7.1)
Other causes	9 (4.3)

*BoNT-A, onabotulinumtoxinA.

### 3.2 Efficacy outcome

In the primary outcome analysis, there was a significant reduction in the headache days after treatment with the BoNT-A (Table 2). With respect the secondary outcome, there was a significant improvement in migraine severity and analgesic days after treatment cycles.

**Table 2:** Primary and second outcomes of BoNT-A treatment on migraine headache patients (*N* = 210).

Primary and secondary outcomes	Migraine state baseline visit M ± SD	Migraine state last visit M ± SD	*P*
Migraine days/months	9.54 ± 1.70	4.58± 2.77	0.001*
Analgesic days/month	8.47 ± 1.49	2.98± 0.21	0.04*
Severity of headache	8.37± 0.72	2.54 ± 0.18	0.001*

*Statistically significant; M, mean; SD, standard deviation.

**Figure 2:**
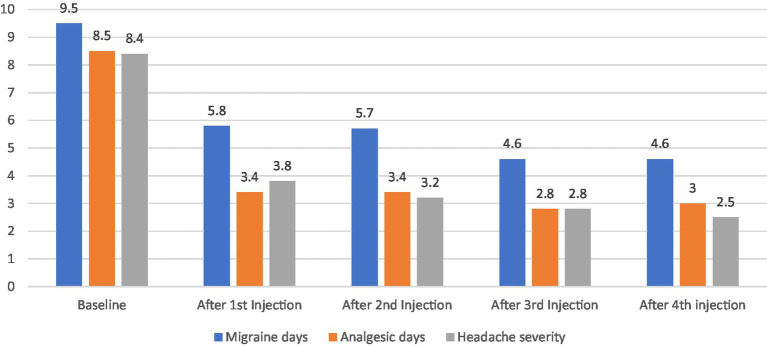
Efficacy and impact of BoNT-A on migraine headache patients (*N* = 210).

Over the 12 months study period, there was a significant reduction in the frequency of migraine days (*p* < 0.001, CI [4.58–5.34]), analgesic days (*p* < 0.047, [3.94–5.05]) and headache severity (*p* < 0.002, [1.44–2.13]) after first BoNT-A injections compared to baseline (30 days prior treatment initiation) and this was maintained over 9 month follow up as illustrated in Figure 2.

At the last visit, after 4 BoNT-A treatment cycles, reduction of migraine headache days and analgesic used days by 50% were reported in 51.4 and 60% of subjects, respectively. However, severity of headache pain improved by 50% among only 21.4% of the total cohort. The satisfaction mean ± SD was 7.19 ± 2.18 after 4 BoNT-A treatment cycles.

### 3.3 Functional outcome

At the last visit BoNT-A treatment improved productivity while working and reduced the impact of headaches on regular daily activities other than work at a job in participants working for pay, as evidenced by lower scores. It had been reported clinical meaningful reduction in missed working hours, ranging from a baseline of (3.23 ± 0.24) per week to (2.24 ± 0.27), *p* < 0.001. In return, working hours increased significantly, where patients reported significant mean changes up to (35.05 ± 0.74) hours per week after treatment with BoNT-A. Table 3 summarizes the impact of BoNT-A treatment on work productivity and activity.

**Table 3:** Secondary outcome: impact of BoNT-A treatment on work productivity and activity.

Variables	Baselines M ± SD/SE	Last visit M ± SD/SE	*P*
Currently employed (working for pay)?			
Yes	147 (86.6)		
No	66 (31.4)		
During the past 7 days, how many hours did you miss from work because of problems associated with your migraine symptoms? Include hours you missed on sick days, times you went in late, left early etc. because of your migraine symptoms. Do not include time you missed to participate in this study?	3.23 ± 2.85/0.24	2.24 ± 3.30/0.27	0.001*
During the past 7 days, how many hours did you miss from work because of any other reason, such as vacation, holidays, time off to participate in this study?	1.42 ± 2.13/0.18	1.31± 2.61/0.22	0.001*
During the past 7 days, how many hours did you work??	31.10 ± 8.10/0.67	35.05± 8.97/0.74	0.001*
During the past 7 days, how much did migraine symptoms affect your productivity while you were working?**	5.62± 1.88/0.15	4.33± 2.58/0.021	0.001*
During the past 7 days, how much did migraine symptoms affect your ability to do your regular daily activities, other than work at a job?***	5.71 ± 2.39/0.12	4.63 ± 2.39/0.17	0.001*

M, mean; SD, standard deviation; SE, standard error. *Statistically significant. **11-point scale: 0 = headache had no effect on work, 10 = headache completely prevented me from working. ***11-point scale: 0 = headache had no effect on daily activities, 10 = headache completely prevented me from daily activities.

Similarly, impact of BoNT-A on MSQ Questionnaire has achieved significance in all criteria as shown in Table 4. Treatment with BoNT-A significantly increased MSQ-RFR scores from baseline compared with last visit, indicating an improvement in patient functioning. Patients with EM showed also significant increases from baseline in the other QoL measurements, MSQ-RFP and MSQ-EF MSQ domain scores compared with last visit. Overall, treatment with BoNT-A resulted in improved QoL, as measured by changes in MSQ-Total. The increase from baseline in MSQ-Total was significantly higher when compared to baseline.

**Table 4:** Impact of BoNT-A on migraine-specific quality of life questionnaire (MSQ).

MSQ	Baseline past 4 weeks M ± SD	Last visit past 4 weeks M± SD	*P*
1. How often have migraines interfered with how well you dealt with family, friends, and others who are close to you?	2.61 ± 0.99	3.90 ± 1.50	0.001*
2. How often have migraines interfered with your leisure time activities, such as reading or exercising?	2.16 ± 1.01	3.51 ± 1.65	0.001*
3. How often have you had difficulty in performing work or daily activities because of migraine symptoms?	2.46 ± 1.17	3.77 ± 1.66	0.001*
4. How often did migraines keep you from getting as much done at work or at home?	2.53 ± 1.31	3.59 ± 1.57	0.001*
5. In the past 4 weeks, how often did migraines limit your ability to concentrate on work or daily activities?	2.36 ± 1.12	3.64 ± 1.66	0.001*
6. How often have migraines left you too tired to do work or daily activities?	2.09 ± 1.04	3.41 ± 1.63	0.001*
7. How often have migraines limited the number of days you have felt energetic?	2.09 ± 0.95	3.29 ± 1.70	0.001*
RFR**	26.89 ± 17.42	51.55 ± 29.12	0.001*
8. How often have you had to cancel work or daily activities because you had a migraine?	2.57 ± 1.02	4.19 ± 1.42	0.001*
9. How often did you need help in handling routine tasks such as everyday household chores, doing necessary business, shopping, or caring for others, when you had a migraine?	2.37 ± 0.95	4.06 ± 1.63	0.001*
10. How often did you have to stop work or daily activities to deal with migraine symptoms?	2.54 ± 1.22	3.70 ± 1.69	0.001*
11. How often were you not able to go to social activities such as parties, dinner with friends, because you had a migraine?	2.65 ± 1.01	3.76 ± 1.53	0.001*
RFP**	30.64 ± 15.25	56.07 ± 24.73	0.001*
12. How often have you felt fed up or frustrated because of your migraines?	2.37 ± 1.05	3.89 ± 1.64	0.001*
13. How often have you felt like you were a burden on others because of your migraines?	3.20 ± 1.43	4.34 ± 1.60	0.001*
14. How often have you been afraid of letting others down because of your migraines?	2.79 ± 1.17	4.31 ± 1.58	0.001*
EF**	35.12 ± 20.83	76.47 ± 115.29	0.001*

*Statistically significant. **Total score of role function restrictive, role function preventive, and emotional function were rescaled from 0 to 100 scale such that higher scores indicate better quality of life. EF, emotional function domain; SE, standard error; M, mean; MSQ, migraine-specific quality-of-life questionnaire; RFP, role function-preventive domain; RFR, role function-restrictive domain; SD: standard deviation.

### 3.4 Safety and tolerability

BoNT-A injections were well tolerated, with no significant or serious AEs were reported. Only 27patients (12.9%) experienced AEs which were mild and short lasting. All of them were females. Ptosis was recorded in 5.7% of patients, while vomiting and dryness was reported similar as low as 1.4%. Neck pain was reported in 4.1%. Treatment discontinuations occurred in 17 (8.1%) patients. Lack of efficacy as persistent of frequent migraine days in 10 (4.8%) and intolerable AEs as ptosis and neck pain 7 (3.3%) were the reported reasons for discontinuation.

## 4 Discussion

According to our research, BoNT-A was a successful preventative treatment for episodic migraine. It dramatically decreased the burden of migraines, the frequency of migraine days, and the use of acute medicines. With BoNT-A treatment, disability and quality of life significantly improved. Our findings aligned with those of a recent open-label, single-arm trial research. It assessed how well BoNT-A prevented high-frequency episodic migraines (21). They followed the same course of treatment as our trial. They only included 32 patients with high frequency EM, but their outcomes—which included fewer migraine days, analgesic drug use, and an improvement in MSQ—were in line with our findings (21).

The effectiveness of BoNT-A in treating episodic migraines has been studied, with varying findings. Silberstein’s findings, which demonstrated that pericranial injection of 25U of BoNT-A dramatically decreased EM frequency, migraine severity, acute medication use, and related vomiting, are comparable to ours (22). Nevertheless, compared to our study and the PREEMPT study, the dose employed in this investigation is lower. The Aurora investigation, which employed three treatment cycles, found a signal of benefit in the subgroup of migraine participants who experienced 12–15 headache days per month, but failed to demonstrate efficacy in episodic migraine (23).

According to our research, after three cycles of BoNT-A treatment, 51.4% of participants reported a 50% decrease in migraine days at 12 months. We found that patients with CM and high frequency EM saw a 41.2% reduction in migraine days at 12 months, which is in line with a prior real-world investigation (24). Compared to Anand’s findings, which showed that 75% of patients with migraine frequency 2–8 months experienced full migraine relief, our data revealed a reduced frequency of migraines (25). Only 32 patients were enrolled in the Anand trial, and they were given 50-U BoNT-A only once. In contrast, Evers’ trial found that 30% of patients experienced a 50% decrease in migraine frequency in month three when compared to baseline (26). The results of our cohort contradict with other real-world data in terms of dosage administered and the efficacy of the prophylaxis of BoNT-A seen in EM patients. Though we have started with 155 units of BoNT-A, according to the PREEMPT paradigm, patients had clearly significant reduction in the mean frequency of migraine episodes per month from baseline. In contrary to Evers et al. (26), migraineurs who were treated with 100U administered with a fixed-site protocol did not result in superior results compared with placebo. This could be attributed to low number of participants, suggesting an underpowered study, and to the location of administration, as patients, in contrast to our study, received either 16U botulinum toxin A in the frontal muscles or 100U botulinum toxin A in the frontal and neck muscles. In our cohort we followed the PREEMPT trial paradigm not the fixed site protocol. Relja et al. (27) evaluated BoNT-A for the prevention of episodic migraine. Although not reaching the primary endpoint, the BoNT-A group had a higher 50% response rate.

Our result showed a significant reduction in the mean frequency of migraine episodes per month (9.54 ± 1.70 vs. 4.58 ± 2.77, *p* < 0.001). In comparison to Relja study, mean reduction in the frequency of migraine episodes per month from baseline was 1.4 in the placebo group vs. 1.5–1.7 episodes in the BoNT-A treated groups. BoNT-A treatment failed to demonstrate a superior effect over placebo group and the primary endpoint was not met (25). The discrepancy in these results could partially explained by a relatively shorter disease duration in our study18.40 ± 10.39 versus 23.5 ± 11.2 in Relja study; since patients become more treatment resistant, as disease duration increases (28).

Although our results show statistical significance efficacy of BoNT-A treatment in reducing the frequency of episodic migraines, similar to other real-world data (29), these findings were contradicted by others. The negative results of Shuhendler et al. (30), who did not find a statistical difference between botulinum toxin type A injection and placebo, had led to the acknowledgment of the inefficacy of botulinum toxin for EM by the American Academy of Neurology in 2008 (31).

The discrepancy between our results and that of the previous results could be explained by the different methods of studies and BoNT-A treatment. Some studies used markedly lower doses of drug, less or different injection sites, different volumes of injections and shorter treatment durations (7, 22–28). Our study used the same paradigm and the same dose that was used for chronic migraine patients in PREEMPT study.

The difference between CM and EM diagnoses is arbitrary. No pathophysiological reason explain that BoNT-A treatment is efficacious in migraine patients with 15 days of headache per month and not effective in people with 14 days of headache per month. BoNT-A treatment may be useful for EM (32).

Improvement observed after 3 months of using BoNT-A in migraine frequency, analgesic days consumption and pain intensity was sustained throughout 1 year follow up. Although treatment with BoNT-A is reported to have a stronger effect as early as 2 months when given for chronic migraines, evidence has shown that effectiveness of the prophylaxis could be shown at month 3 in episodic migraine, which was not previously emphasized (30, 33). This suggests that even when BoNT-A was used in smaller dosage 25U (but not 75U), it significantly reduced the frequency of moderate-to-severe migraine episodes from baseline compared with placebo at months 2 (1.6 vs. 0.4, *p* = 0.008) and 3 (1.9 vs. 1.0, *p* = 0.042) (22).

BoNT-A improved work productivity and reduced the mean number of hours missed from work over a 7-day period. This study, BoNT-A treatment resulted in significantly reductions in headache-related work productivity loss as measured by WPAI compared with the baseline before treatment. Our result agreed with Blumenfeld study that reported that BoNT-A treatment had more favorable real-world effectiveness than topiramate on daily living, activity, and work productivity (34).

Patients with EM receiving BoNT-A for at least 4 cycles showed improvements in the RFR, RFP, and EF domains of the MSQ relative to base line. Similarly, improvements in MSQ domain scores in patients with migraine have also been reported in previous studies that revealed significant improvements in MSQ role-restrictive, role-preventive, and emotional-functioning domain scores favoring BoNT-A over placebo (35, 36). Similarly, results from the open-label REPOSE study revealed improvements over a 2-year period in migraine-specific quality of life, assessed using the MSQ Questionnaire (7).

The incidence of AEs in our study were similar to the incidence reported in the previous pivotal studies (11, 12) and real-world studies (30–33, 37). Reported AE in this study were mild and transient.

### 4.1 Limitations of the study

This study had several limitations, which should be considered when interpreting the results. The main limitation was that the patients were not blinded to the BoNT-A therapy. Second, there was no placebo group or an active comparator. Third, a long-term treatment beyond 1 year was not evaluated in this cohort. We did simple statistical analysis. We did not study confounder as gender, hormonal change, socioeconomic status, other medications used, and comorbidities. Finally, the use of self-reported data was subject to recall bias and information bias. The WPAI has not been validated in migraine.

### 4.2 Strengths of the study

The strengths of the present study included the fact that this was first study to assess BoNT-A therapy for EM in the Middle East. It included relatively a reasonable sample size. The use of MSQ which is a migraine-specific tool, unlike more general QoL scales, give more credibility to the study.

We recommend having further study included larger cohort for longer duration of follow up with sophisticated statistical analysis.

## 5 Conclusion

Strict adherence to PREEMT protocol in this study favored the BoNT-A treatment in EM. BoNT-A treatment reduced monthly migraine days and acute medication use. Reduction of monthly migraine days and analgesic consumption were maintained throughout the one-year study period. BoNT-A treatment improved quality of life and work productivity, supporting the benefits of using BoNT-A for EM. BoNT-A treatment was well tolerated and safe. We suggest that BoNT-A should be considered in patients with high frequency episodic migraine to prevent transformation to chronic migraine which is more difficult to treat.

## Data Availability

The raw data supporting the conclusions of this article will be made available by the authors, without undue reservation.
